# Systemic therapy following craniotomy in patients with a solitary breast cancer brain metastasis

**DOI:** 10.1007/s10549-020-05531-7

**Published:** 2020-01-17

**Authors:** Alexander F. C. Hulsbergen, Logan D. Cho, Marco Mammi, Nayan Lamba, Timothy R. Smith, Priscilla K. Brastianos, Marike L. D. Broekman, Nancy U. Lin

**Affiliations:** 1 Computational Neuroscience Outcomes Center, Department of Neurosurgery, Brigham & Women’s Hospital, Harvard Medical School, 75 Francis Street, Boston, MA 02115 USA; 2 Faculty of Medicine, Utrecht University, Universiteitsweg 98, 3584 CG Utrecht, The Netherlands; 3 Department of Neurosurgery, Leiden University Medical Center, Zuid-Holland, Albinusdreef 2, 2300 RC Leiden, The Netherlands; 4 Department of Neurosurgery, Haaglanden Medical Center, Lijnbaan 32, 2512 VA The Hague, The Netherlands; 5 Brown University, 69 Brown Street, Providence, RI 02912 USA; 6 Department of Medicine, Massachusetts General Hospital, 55 Fruit Street, Boston, MA 02114 USA; 7 Department of Neurology, Massachusetts General Hospital, 55 Fruit Street, Boston, MA 02114 USA; 8 Division of Breast Oncology, Department of Medical Oncology, Dana-Farber Cancer Institute, Harvard Medical School, 450 Brookline Avenue, Boston, MA 02215 USA

**Keywords:** Breast cancer, Solitary brain metastasis, Craniotomy, Systemic therapy, Hormonal therapy, HER2-targeted therapy

## Abstract

**Purpose:**

To describe practice patterns and patient outcomes with respect to the use of postoperative systemic therapy (ST) after resection of a solitary breast cancer brain metastasis (BCBM).

**Methods:**

A multi-institutional retrospective review of consecutive patients undergoing resection of a single BCBM without extracranial metastases was performed to describe subtype-specific postoperative outcomes and assess the impact of types of ST on site of recurrence, progression-free survival (PFS), and overall survival (OS).

**Results:**

Forty-four patients were identified. Stratified estimated survival was 15, 24, and 23 months for patients with triple negative, estrogen receptor positive (ER+), and HER2+ BCBMs, respectively. Patients receiving postoperative ST had a longer median PFS (8 versus 4 months, adjusted *p*-value 0.01) and OS (32 versus 15 months, adjusted *p*-value 0.21). Nine patients (20%) had extracranial progression, 23 (52%) had intracranial progression, three (8%) had both, and nine (20%) did not experience progression at last follow-up. Multivariate analysis showed that postoperative hormonal therapy was associated with longer OS (HR 0.26; 95% CI 0.08–0.89; *p* = 0.03) but not PFS (HR 0.35, 95% CI 0.08–1.47, *p* = 0.15) in ER+ patients. Postoperative HER2-targeted therapy was not associated with longer OS or PFS in HER2+ patients.

**Conclusions:**

Disease progression occurred intracranially more often than extracranially following resection of a solitary BCBM. In ER+ patients, postoperative hormonal therapy was associated with longer OS. Postoperative HER2-targeted therapy did not show survival benefit in HER2+ patients. These results should be validated in larger cohorts.

## Introduction

Brain metastases are the most common intracranial tumors and frequently originate from lung cancer, melanoma, or breast cancer [1, 2]. The prognosis of patients with breast cancer brain metastases (BCBM) has historically been poor, but advances in systemic therapy (ST) have prolonged survival primarily by providing better extracranial control [3, 4]. However, the blood–brain barrier (BBB) denies many systemic agents optimal access to the central nervous system (CNS), creating a sanctuary site for distant metastases [5]. This has led to an increase in the incidence of BMs, including those in the absence of extracranial disease [6, 7].

Standard treatment for solitary BMs consists of surgical resection followed by adjuvant radiation, either stereotactic radiosurgery (SRS), whole-brain radiotherapy (WBRT), or a combination [8, 9]. There are no systemic treatments specifically approved for the purpose of BCBM, although some patients still receive systemic agents in the context of clinical trials, for extracranial metastases, or as off-label use. In the absence of extracranial metastases, there is considerable practice variation in the administration of postoperative ST for these patients, due to a lack of data demonstrating benefit [10].

Little is known about the efficacy of postoperative systemic therapy in breast cancer patients who present with a BM. While increased survival in systemically treated patients with solitary BMs has been reported, these findings have not been validated, especially in the neurosurgical population [11, 12]. Moreover, the extent to which chemotherapy, hormonal therapy, and/or human epidermal growth factor receptor 2 (HER2)-targeted therapy can prevent intra- or extracranial recurrence after neurosurgical resection is unknown. Due to insufficient evidence, most guidelines have either not recommended or not commented upon the use of ST after BM resection, when performed in the setting of CNS-only disease [13]. The goals of the present study were to describe practice patterns regarding the use of postoperative ST and to explore correlations between type of systemic therapy and site of recurrence, progression-free survival (PFS), and overall survival (OS) in patients who underwent surgical resection for a solitary BM as the first site of metastatic breast cancer.

## Methods

Under Institutional Review Board approval, a retrospective review of electronic patient records of the Brigham and Women’s Hospital and Massachusetts General Hospital was conducted. Female patients who underwent neurosurgical resection of a newly diagnosed solitary BM of breast cancer between January 1, 2002 and August 1, 2017 were included. A solitary BM was defined as one brain metastasis with no previous or concomitant intracranial or extracranial metastasis. Patients who had diffuse leptomeningeal enhancement at the time of resection were included. Exclusion criteria were uncertainty about primary origin of the BM, lack of follow-up, and appearance of new metastases between the point of craniotomy and the first postoperative visit to the medical oncologist. Patients were not excluded if they had ambiguous radiographic findings not directly suspicious of metastasis (e.g., non-specific diffuse leptomeningeal enhancement or diffuse lymphadenopathy).

Data were collected on demographics, breast cancer subtype, date of BM surgery, extent of resection (gross total versus subtotal as determined on postoperative MRI), dexamethasone use, adjuvant SRS and WBRT, pre- and postoperative systemic treatments, date and location of disease progression, and date of death or last follow-up. Breast cancer subtype was based on expression of estrogen (ER) and HER2 status of the brain lesion and classified as ER+/HER2+, ER+/HER2−, ER−/HER2+, and ER−/HER2−. ER was considered positive if > 10% of nuclei showed positive immunohistochemistry (IHC) staining. HER2 was considered positive if IHC revealed strong (3+) overexpression or moderate (2+) overexpression with amplification in fluorescence in situ hybridization (HER2/centromere ratio > 2.0). Localization of disease recurrence, PFS, and OS were the primary outcomes of this study. Survival was defined as the interval from date of BM resection to date of progression or last follow-up for PFS, and date of death or last follow-up for OS. Date of progression was defined as the day of initial radiological appearance of progression. Patients who had no available date of death were censored at date of last encounter, with a study cut-off date of August 1, 2018.

### Statistical analysis

Data were analyzed using R version 3.4.3 (R Foundation for Statistical Computing, Vienna, Austria). Descriptive statistics were used to express demographics and clinical characteristics. Categorical variables were expressed as counts and percentages. Continuous variables were reported using mean and standard deviation if they followed a normal distribution, or median and interquartile range (IQR) if they did not. Efficacy of systemic therapy was analyzed in the following populations: chemotherapy for the entire cohort, hormonal therapy for ER+ patients, and HER2-targeted therapy for HER2+ patients. Univariate and multivariate survival analyses were performed using the Cox Proportional Hazards Model, and statistical significance was determined with the Log-rank test. The threshold for significance was set at *p* < 0.1 when screening for potential confounders in univariate analysis and at *p* < 0.05 to determine impact on survival in multivariate analysis. The multivariate model incorporated systemic therapy as well as breast cancer subtype, age, and type of adjuvant radiation; lastly any potential confounders identified in univariate analysis were also included. Kaplan–Meier curves generated to visualize differences in survival between subtypes and treatment modalities.

## Results

### Descriptive statistics

Out of 196 patients with resected BCBMs, a total of 44 fitted inclusion criteria. Patient characteristics are summarized in Table 1. The median age was 55 years (IQR 47.5–61.3 years). When stratified by subtype, thirteen (30%) patients were ER−/HER2−, nine (20%) were ER-/ HER2+, sixteen (36%) were ER+/HER2−, and six (14%) were ER+/HER2+. Of the 44 patients, 35 (80%) received postoperative dexamethasone. Adjuvant radiation consisted of whole WBRT (*n* = 21; 48%), SRS (*n* = 17, 39%) or hypofractionated stereotactic radiotherapy (HFSRT; *n* = 6; 14.6%). Twenty-two patients (50%) received some form of ST, with four (9%) patients receiving chemotherapy, eight (18%) receiving HER2-targeted therapy, and twelve (27%) receiving hormonal therapy. At the time of data-lock, 27 patients (61%) had died. The median PFS of the entire cohort was 5.3 months (IQR 2.8–17.5 months) and the median OS was 23.9 months (IQR 14.1–66.5 months).

**Table 1:** Patient characteristics stratified by breast cancer subtype

Subtype	ER−/HER2−(%)	ER−/HER2+ (%)	ER+/HER2− (%)	ER+/HER2+ (%)	Total (%)
*N*	13	9	16	6	44
Race					
Asian	1 (7.7)	0 (0.0)	1 (6.2)	1 (16.7)	3 (6.8)
Black	1 (7.7)	1 (11.1)	0 (0.0)	1 (16.7)	3 (6.8)
Hispanic	1 (7.7)	0 (0.0)	0 (0.0)	0 (0.0)	1 (2.3)
Other	0 (0.0)	2 (22.2)	1 (6.2)	0 (0.0)	3 (6.8)
Unknown	0 (0.0)	0 (0.0)	0 (0.0)	1 (16.7)	1 (2.3)
White	10 (76.9)	6 (66.7)	14 (87.5)	3 (50.0)	33 (75.0)
Age at NSG [mean (SD)]	59.2 (8.3)	53.3 (10.3)	57.4 (15.0)	45.7 (10.8)	55.5 (12.3)
Disease free interval in months [mean (SD)]	16.2 (13.2)	54.2 (108.0)	32.9 (26.2)	50.0 (36.7)	34.7(53.2)
History of systemic therapy before NSG					
Chemotherapy	13 (100.0)	9 (100.0)	14 (87.5)	3 (60.0)	39 (90.7)
Hormonal therapy	1 (7.7)	0 (0.0)	11 (68.8)	6 (100.0)	18 (40.9)
HER2-targeted therapy	0 (0.0)	6 (66.7)	0 (0.0)	5 (83.3)	11 (25.6)
Other systemic therapy	3 (23.1)	0 (0.0)	2 (12.5)	1 (25.0)	6 (14.6)
Extent of resection					
Gross total	10 (76.9)	7 (77.8)	13 (81.2)	4 (66.7)	34 (77.3)
Subtotal	2 (15.4)	2 (22.2)	3 (18.8)	2 (33.3)	9 (20.5)
Undetermined	1 (7.7)	0 (0.0)	0 (0.0)	0 (0.0)	1 (2.3)
Postoperative BM-directed treatment					
Dexamethasone	10 (76.9)	6 (66.7)	13 (81.2)	6 (100.0)	35 (79.5)
WBRT	5 (38.5)	2 (22.2)	11 (68.8)	3 (50.0)	21 (47.7)
SRS	6 (46.2)	6 (66.7)	3 (18.8)	2 (33.3)	17 (38.6)
HFSRT	2 (15.4)	1 (11.1)	2 (12.5)	1 (16.7)	6 (14.6)
Postoperative systemic therapy	2 (15.4)	6 (66.7)	9 (56.2)	5 (83.3)	22 (50.0)
Chemotherapy	1 (7.7)	2 (22.2)	1 (6.2)	0 (0.0)	4 (9.1)
Hormonal therapy^a^	1 (7.7)	0 (0.0)	8 (50.0)	3 (50.0)	12 (27.3)
HER2-targeted therapy^b^	0 (0.0)	5 (55.6)	0 (0.0)	3 (50.0)	8 (18.2)
Other systemic therapy	1 (7.7)	0 (0.0)	0 (0.0)	0 (0.0)	1 (2.4)

*BM* brain metastasis, *ER* estrogen receptor, *HER2* human epidermal growth factor receptor 2, *HFSRT* Hypofractionated stereotactic radiotherapy, *NSG* neurosurgery, *SD* standard deviation, *SRS* stereotactic radiosurgery, *WBRT* whole-brain radiotherapy

^a^Hormonal therapy consisted of tamoxifen (*n* = 4), aromatase inhibitors (*n* = 6), or others (*n* = 2)

^b^HER2 therapy consisted of trastuzumab (*n* = 6), lapatinib (*n* = 1), or both (*n* = 1)

**Fig. 1:**
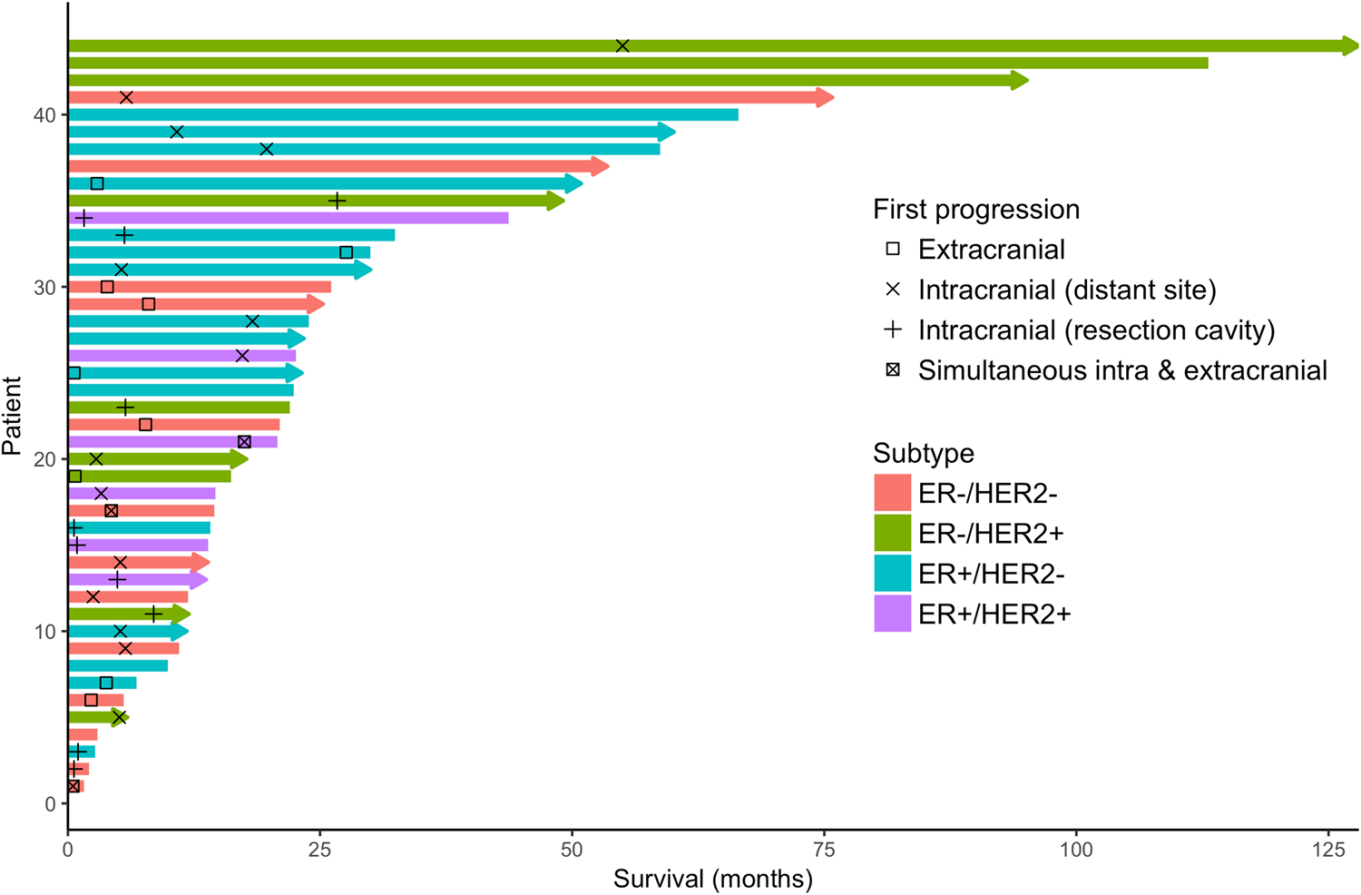
Swimmer’s plot showing recurrence patterns and survival by subtype. *ER* estrogen receptor, *HER2* human epidermal growth factor receptor 2. Arrows at the end of a bar indicate that the patient was still alive after the date of last follow-up

**Fig. 2:**
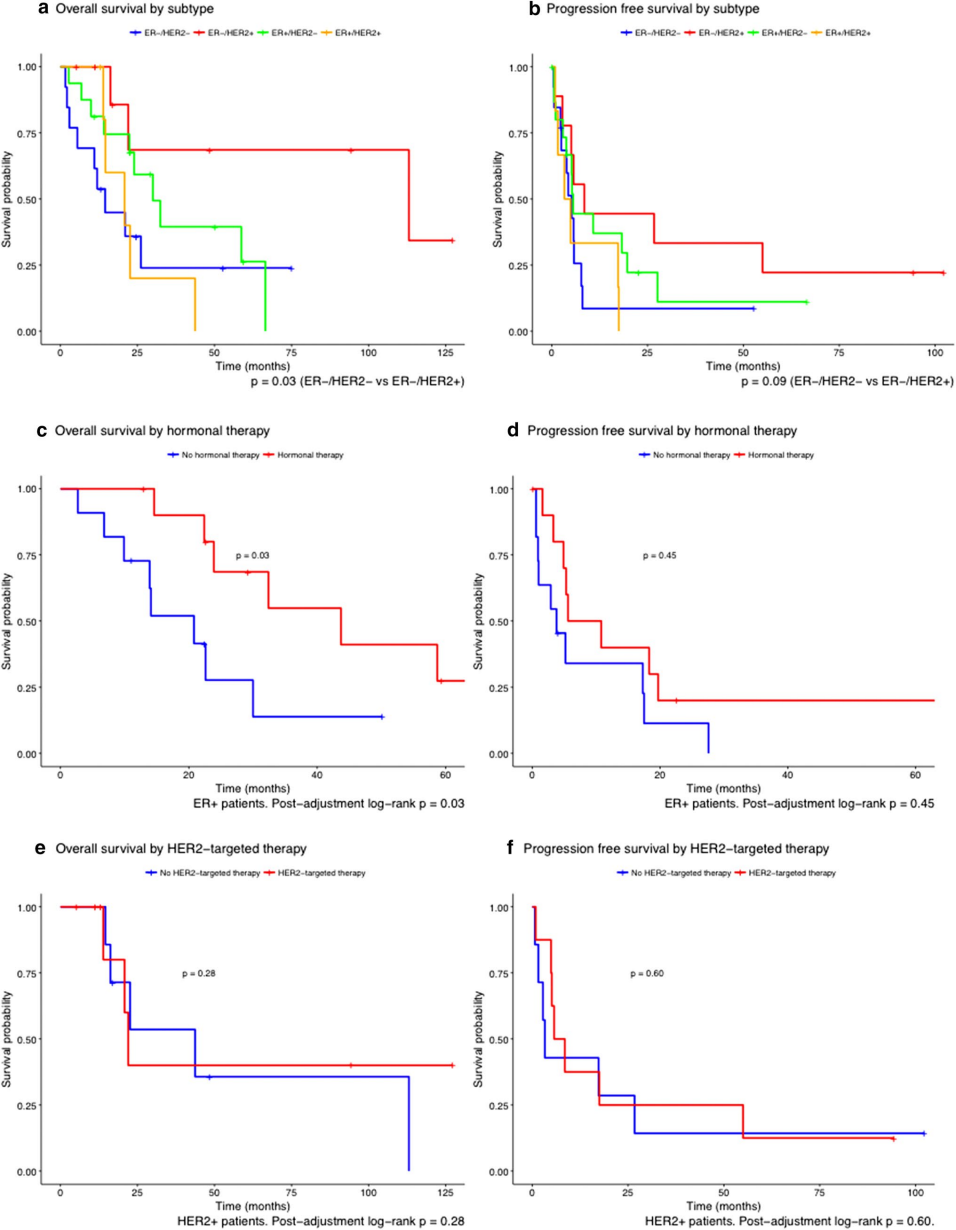
Kaplan–Meier curves showing overall and progression-free survival by **a**, **b** subtype, **c**, **d** hormonal therapy in ER+ patients, and **e**, **f** HER2-targeted therapy in HER2+ patients. *ER* estrogen receptor, *HER2* human epidermal growth factor receptor 2

Outcomes stratified by administration of ST are described in Table 2. First site of progression following surgery was extracranial in nine patients (20%) and intracranial in 24 patients (55%), of which ten (23%) experienced local recurrence and fourteen (32%) had a recurrence at a distant site in the brain. Three patients (7%) presented with both intra- and extracranial progression simultaneously during follow-up. The remaining eight patients (18%) did not show signs of progression at their last follow-up. Patients treated with ST had longer PFS (8.0 vs 3.9 months) and OS (32.4 vs 14.5 months) compared to patients who did not receive ST, although this was not statistically significant (unadjusted *p* = 0.14 and *p* = 0.13, respectively). Because this comparison could be influenced by the fact that more triple-negative patients were in the no-ST group, we present OS and PFS stratified by subtype and presence/absence of ST in Table 3. Median PFS was 5.2, 5.3, and 5.7 months for ER−/HER2−, ER+, and HER2+ patients, respectively. Median OS was 14.5, 23.9, and 22.6 months for ER−/HER2−, ER+, and HER2+ patients, respectively. All subtypes showed a longer PFS in the ST group, while only ER+ patients treated with ST had a longer OS (Table 3). Figure 1 displays patterns of recurrence and survival by subtype and systemic treatment on a per-patient level. Kaplan–Meier curves of OS and PFS by subtype are displayed in Fig. 2a and b.

**Table 2:** Patterns of progression and survival based on administration of postoperative systemic therapy

	Postoperative systemic therapy	No postoperative systemic therapy	*p*-value
*N*	22	22	
Location of first Progression (%)			0.73
Extracranial	3 (13.6)	6 (27.3)	
Intracranial	14 (63.6)	10 (45.5)	
Local	6 (27.3)	4 (18.2)	
Distant	8 (36.4)	6 (27.3)	
Both recorded simultaneously	1 (4.5)	2 (9.1)	
No recorded progression	4 (18.2)	4 (18.2)	
Progression-free survival in months (median[IQR])	8.0 [5.1, 19.7]	3.9 [2.3, 7.7]	0.14
Overall survival in months (median[IQR])	32.4 [22.0, 66.5]	14.5 [6.8, 113.1]	0.13

*IQR* interquartile range, *NR* not reached

**Table 3:** Median progression-free survival and overall survival by receptor status and receipt of subtype-specific systemic therapy

Subtype	Systemic therapy^a^	No systemic therapy	Total
Progression-free survival			
ER−/HER2− (*n* = 13)	8.1	4.3	5.2
ER+ (*n* = 22)	8.2	3.8	5.3
HER2+ (*n* = 15)	7.1	3.3	5.7
Total (*n* = 44)	8.0	3.9	5.3
Overall survival			
ER−/HER2− (*n* = 13)	NR	13.2	14.5
ER+ (*n* = 22)	43.7	20.8	23.9
HER2+ (*n* = 15)	22.0	43.7	22.6
Total	32.4	14.5	23.9

Descriptive statistics on median OS and PFS times by receipt of systemic therapy (HER2-targeted therapy in HER2+ patients, hormonal therapy in ER+ patients, and chemotherapy in triple-negative patients). ER+/HER2+ patients are included in both ER+ and HER2+ subgroups

*ER* estrogen receptor, *HER2* human epidermal growth factor receptor 2, *NR* not reached

^a^Only one patient in the triple-negative group received postoperative chemotherapy; this patient was alive after 25 months of follow-up

### Survival analysis

To adjust for potential confounders, multivariate survival analyses were performed for both PFS and OS. For PFS, more recent year of treatment (HR 1.08 for every subsequent year; 95% CI 1.00–1.18; *p* = 0.046) was prognostically unfavorable; this variable was included into the multivariate model. For OS, no potential confounders were identified in univariate analysis.

In the multivariate model for PFS, patients who received postoperative ST had a superior PFS to those who did not (HR 0.33; 95% CI 0.14–0.76; *p* = 0.01) after adjusting for subtype, age, year of treatment, and type of adjuvant radiation. ER−/HER2+ subtype was also associated with a trend towards improved survival when compared against triple-negative subtype (HR 0.35, 95% CI 0.11–1.09, *p* = 0.07). In the multivariate model for OS, ER−/HER2+ subtype (HR 0.24; 95% CI 0.05–1.18; *p* = 0.08) showed a trend for improved OS. The receipt of postoperative ST was not significantly correlated with OS (HR 0.55; 95% CI 0.21–1.39; *p* = 0.21).

Next, we stratified survival analysis by type of systemic therapy. In ER+ patients, postoperative hormonal therapy was associated with longer OS (HR 0.30; 95% CI 0.09–0.93; *p* = 0.04) but not PFS (HR 0.50; CI 0.19–1.29; *p* = 0.15, Fig. 2c and d) in univariate analysis. No further potential confounders were identified. After adjustment for age, subtype, and adjuvant radiotherapy in multivariate analysis, postoperative hormonal therapy was prognostically favorable for OS (HR 0.25; 95% CI 0.07–0.89; *p* = 0.03), but not PFS (HR 0.35, 95% CI 0.08–1.47, *p* = 0.15). In HER2+ patients, postoperative HER2-targeted therapy was not significantly associated with OS (HR 0.83; 95% CI 0.20–3.52; *p* = 0.81) or PFS (HR 0.80; 95% CI 0.27–2.41; *p* = 0.69, Fig. 2e and f) in univariate analysis. No other potential confounders were identified. These results did not change after covariate adjustment in multivariate analysis (OS: HR 1.88, 95% CI 0.21–4.55, *p* = 0.57; PFS: HR 0.26, 95% CI 0.05–1.32, *p* = 0.10). Since only four patients received postoperative chemotherapy, we were unable to meaningfully analyze the effects of this treatment on PFS and OS.

## Discussion

This retrospective, multi-institutional study analyzed the outcomes of breast cancer patients after resection of a solitary BCBM and explored the impact of postoperative ST strategies on PFS and OS. In this specific population, PFS was 5.3 months and OS was 23.9 months after neurosurgical resection. In approximately half of patients, the brain was the first site of subsequent progression, whereas extracranial progression was the first site of subsequent progression in only 20% of patients. Administration of postoperative hormonal therapy was associated with improved OS in ER+ patients, while postoperative HER2-targeted therapy showed no correlation with outcomes in HER2+ patients. To our knowledge, this is the first study to assess the role of targeted systemic therapies on survival in breast cancer patients stratified by receptor subtype following resection of a solitary BCBM.

Since the seminal studies by Patchell et al. and Vecht et al., surgical resection has been considered standard of care for patients presenting with a single brain metastasis [14, 15]. In these studies, which primarily included patients with primary lung cancer, and only a small proportion of breast cancer patients, median survival was between 9–10 months in the surgical group. In our patient population, median OS was 23.9 months. This was driven primarily by patients with ER+ or HER2+ tumors; those with triple-negative tumors experienced median OS of only 14.5 months, even though we restricted our population to those who had no evidence of extracranial disease involvement at the time of neurosurgical resection. Among 9 patients with ER−/HER2+ tumors, 4 (44%) were alive and progression-free two years after surgical resection; whereas this was the case for only 2/16 patients with ER+/HER2− subtype and only 1/12 patients with ER−/HER2− subtype. Thus, a subset of patients may experience extended disease control and survival despite a diagnosis of brain metastasis. However, patients with triple-negative breast cancer still experience significantly worse outcomes, even when selecting for the most favorable patients (i.e., a resectable, single brain lesion in the absence of extracranial involvement).

In terms of the impact of ST, we found that patients who received postoperative ST experienced longer PFS after adjusting for confounders (HR 0.33; *p* = 0.01). When stratifying by type of systemic therapy, hazard ratios did suggest a favorable survival associated with hormonal therapy in ER+ patients (HR 0.54, *p* = 0.25) or HER2-targeted therapy in HER2+ patients (HR 0.26, *p* = 0.10), but this did not reach statistical significance. This could in part be due to a smaller sample size in stratified analysis leading to diminished statistical power. In terms of OS, postoperative hormonal therapy was associated with favorable survival in hormonal patients (HR 0.26, *p* = 0.03). Somewhat surprisingly, we were not able to detect any associations between postoperative HER2-targeted therapy and OS (HR 0.83; *p* = 0.81). Taken together, these results could indicate that postoperative systemic therapy is associated with better PFS, which seems to be irrespective of type of systemic therapy. In ER+ patients, hormonal therapy may also confer an OS benefit after solitary BCBM resection.

Our results lie in line with previous literature. In a 2011 prospective series, ER/PR + breast cancer patients with a single BCBM were treated with WBRT and approximately two-thirds went on to receive ST [12]. The authors reported that treatment with WBRT + ST was associated with increased survival when compared to WBRT alone [12]. Subsequently, the authors of the study began to prescribe systemic treatment for nearly all patients with a brain metastasis as the first and only location of recurrence [11]. They observed that systemic therapy was associated with prolonged survival for patients with a single BCBM and no extracranial metastases [11]. Unlike in our study, not all the patients in this trial underwent surgical resection prior to receiving systemic therapy.

Our study adds to the prior literature that suggests a survival benefit for systemic therapy in patients with BCBM by focusing specifically on patients who have undergone resection. Moreover, the previously published study did not stratify patients by type of systemic therapy. We stratified breast cancer patients by tumor receptor subtype and demonstrated a correlation between receipt of hormonal therapy and prolonged survival in patients with ER+ breast cancer specifically.

In vitro studies have demonstrated that tamoxifen and the aromatase inhibitors letrozole and vorozole can easily cross the BBB, while anastrazole does so to a lesser extent [16, 17]. Multiple case reports document responses of BCBMs to tamoxifen and aromatase inhibitors [18–21]. It is thus biologically plausible that postoperative hormonal therapy could treat micrometastatic disease. Given the favorable toxicity profile of hormonal therapy, we believe that our results, though based upon a small sample size, would support the use of hormonal therapy after resection of a solitary brain metastasis in patients with ER+ breast cancer. Of note, our study predated the widespread use of CDK4/6 inhibitors, some of which penetrate the BBB and have demonstrated preliminary evidence of efficacy against progressive brain metastases [22]. Future studies evaluating the role of CDK4/6 inhibitors in the postoperative setting may thus be of interest.

In contrast, in our study, HER2-targeted therapy was not associated with improved survival in patients with HER2+ tumors. We acknowledge we cannot fully rule out a potential benefit due to our small sample size. In addition, it is unknown whether the newer generation of HER2-targeted tyrosine kinase inhibitors, such as neratinib or tucatinib, might have a beneficial effect in this setting. Nevertheless, we believe our data may be useful in discussing the risks and benefits of postoperative anti-HER2 therapy with patients. Because only four patients received postoperative chemotherapy, we were unable to meaningfully analyze the effects of this treatment on PFS and OS. In the setting of more significant potential toxicities with chemotherapy (as compared with hormonal therapy or HER2-directed therapy), we believe it would be difficult to justify the routine use of postoperative chemotherapy without more data.

### Strengths and limitations

The primary strength of this study is its homogenous, clearly defined patient population. Because all patients had a newly diagnosed BCBM as the first and isolated site of recurrence, we avoided heterogeneity with respect to previous lines of systemic treatment for metastatic breast cancer, which could have confounded analysis. Moreover, we were able to base subtype off receptor status in the BM rather than the primary tumor. Hormonal and HER2 receptor expression can differ between primary and (neuro)metastatic breast cancer, with loss of hormonal receptor status being the most common discordance [23]. By taking into account BM subtype, we were able to control for loss of receptor status and predicted susceptibility to hormonal therapy.

The size of the present cohort presents the main limitation. Most surgical BCBM patients presented with some extracranial involvement either at the time of resection or between resection and the first postoperative visit to the medical oncologist, which was usually about a month later. While a larger sample might have made for stronger statistical analysis, the homogeneity of the cohort was necessary to analyze this specific clinical question. We observed some trends that approached but failed to reach statistical significance, possibly because we were underpowered to detect such differences. While a significant survival advantage of hormonal therapy in ER+ patients was discernable, our analyses should be replicated in a larger sample size. Lastly, as this is a retrospective study, conclusions must be carefully extrapolated. The effect of systemic interventions on outcomes would ideally be determined in a prospective randomized trial.

Despite these limitations, we believe our study addresses a gap in the current literature by providing data that could be useful in patient-physician discussions regarding anticipated risks, benefits, and outcomes of surgical resection in patients with breast cancer, in a subtype-specific manner.

## Conclusions

In this multi-institutional, retrospective review of patients who underwent resection for a solitary BCBM, we describe practice patterns and survival/recurrence outcomes by administration of postoperative systemic therapy in the absence of extracranial disease. The majority of disease recurrences in this population were intracranial. In ER+ patients, postoperative hormonal therapy was significantly associated with longer OS. We were not able to detect any associations between postoperative HER2-targeted therapy and PFS or OS in HER2+ patients, though our findings must be interpreted with caution due to small sample size. Further studies pooling larger datasets and/or multicenter prospective studies could provide further clarity on the role of postoperative systemic therapy in this patient population.

## References

[1] Ferlay J, Soerjomataram I, Dikshit R, Eser S, Mathers C, Rebelo M, Parkin DM, Forman D, Bray F (2015). Cancer incidence and mortality worldwide: sources, methods and major patterns in GLOBOCAN 2012. Int J Cancer.

[2] Barnholtz-Sloan JS, Sloan AE, Davis FG, Vigneau FD, Lai P, Sawaya RE (2004). Incidence proportions of brain metastases in patients diagnosed (1973 to 2001) in the metropolitan detroit cancer surveillance system. J Clin Oncol.

[3] Lin NU (2013). Breast cancer brain metastases: new directions in systemic therapy. Ecancermedicalscience.

[4] Fontanella C, De Carlo E, Cinausero M, Pelizzari G, Venuti I, Puglisi F (2016). Central nervous system involvement in breast cancer patients: is the therapeutic landscape changing too slowly?. Cancer Treat Rev.

[5] Boaziz C, Breau JL, Morere JF, Israel L (1991). The blood–brain barrier: implications for chemotherapy in brain tumors. Pathol Biol.

[6] Olson EM, Abdel-Rasoul M, Maly J, Wu CS, Lin NU, Shapiro CL (2013). Incidence and risk of central nervous system metastases as site of first recurrence in patients with HER2-positive breast cancer treated with adjuvant trastuzumab. Ann Oncol.

[7] Kodack DP, Askoxylakis V, Ferraro GB, Fukumura D, Jain RK (2015). Emerging strategies for treating brain metastases from breast cancer. Cancer Cell.

[8] Lamba N, Muskens IS, DiRisio AC, Meijer L, Briceno V, Edrees H, Aslam B, Minhas S, Verhoeff JJC, Kleynen CE, Smith TR, Mekary RA, Broekman ML (2017). Stereotactic radiosurgery versus whole-brain radiotherapy after intracranial metastasis resection: a systematic review and meta-analysis. Radiat Oncol.

[9] Liu Y, Alexander BM, Chen YH, Horvath MC, Aizer AA, Claus EB, Dunn IF, Golby AJ, Johnson MD, Friesen S, Mannarino EG, Wagar M, Hacker FL, Arvold ND (2015). Salvage whole brain radiotherapy or stereotactic radiosurgery after initial stereotactic radiosurgery for 1–4 brain metastases. J Neuro-oncol.

[10] Lin NU, Gaspar LE, Soffietti R (2017). Breast cancer in the central nervous system: multidisciplinary considerations and management. Am Soc Clin Oncol Educ Book.

[11] Niwinska A (2016). Brain metastases as site of first and isolated recurrence of breast cancer: the role of systemic therapy after local treatment. Clin Exp Metastasis.

[12] Niwinska A, Pogoda K, Murawska M, Niwinski P (2011). Factors influencing survival in patients with breast cancer and single or solitary brain metastasis. Eur J Surg Oncol.

[13] Ramakrishna N, Temin S, Chandarlapaty S, Crews JR, Davidson NE, Esteva FJ, Giordano SH, Kirshner JJ, Krop IE, Levinson J, Modi S, Patt DA, Perlmutter J, Winer EP, Lin NU (2018). Recommendations on disease management for patients with advanced human epidermal growth factor receptor 2-positive breast cancer and brain metastases: ASCO clinical practice guideline update. J Clin Oncol.

[14] Patchell RA, Tibbs PA, Walsh JW, Dempsey RJ, Maruyama Y, Kryscio RJ, Markesbery WR, Macdonald JS, Young B (1990). A randomized trial of surgery in the treatment of single metastases to the brain. N Engl J Med.

[15] Vecht CJ, Haaxma-Reiche H, Noordijk EM, Padberg GW, Voormolen JH, Hoekstra FH, Tans JT, Lambooij N, Metsaars JA, Wattendorff AR (1993). Treatment of single brain metastasis: radiotherapy alone or combined with neurosurgery?. Ann Neurol.

[16] Miyajima M, Kusuhara H, Takahashi K, Takashima T, Hosoya T, Watanabe Y, Sugiyama Y (2013). Investigation of the effect of active efflux at the blood–brain barrier on the distribution of nonsteroidal aromatase inhibitors in the central nervous system. J Pharm Sci.

[17] Lien EA, Wester K, Lonning PE, Solheim E, Ueland PM (1991). Distribution of tamoxifen and metabolites into brain tissue and brain metastases in breast cancer patients. Br J Cancer.

[18] Madhup R, Kirti S, Bhatt ML, Srivastava PK, Srivastava M, Kumar S (2006). Letrozole for brain and scalp metastases from breast cancer: a case report. Breast.

[19] Tural D, Akar E, Sager S, Yildiz O, Ozguroglu M (2013). A case of complete response to letrozol treatment in a postmenopausal woman with breast cancer who has progressed after multiple lines of chemotherapy. World J Oncol.

[20] Ito K, Ito T, Okada T, Watanabe T, Gomi K, Kanai T, Mochizuki Y, Amano J (2009). A case of brain metastases from breast cancer that responded to anastrozole monotherapy. Breast J.

[21] Goyal S, Puri T, Julka PK, Rath GK (2008). Excellent response to letrozole in brain metastases from breast cancer. Acta Neurochir.

[22] Hurvitz SA, Gelmon KA, Tolaney SM (2017). Optimal management of early and advanced HER2 breast cancer. Am Soc Clin Oncol Educ Book.

[23] Schrijver W, Suijkerbuijk KPM, van Gils CH, van der Wall E, Moelans CB, van Diest PJ (2018). Receptor conversion in distant breast cancer metastases: a systematic review and meta-analysis. J Natl Cancer Inst.

